# Supplementary material for: Lociq provides a loci-seeking approach for enhanced plasmid subtyping and structural characterization
Source: Commun Biol. 2023 Jun 2;6:595. doi: 10.1038/s42003-023-04981-1 (PMC10238380; doi:10.1038/s42003-023-04981-1)
Supplement: Supplementary file 2 — Supplementary Information [file 42003_2023_4981_MOESM2_ESM.pdf]

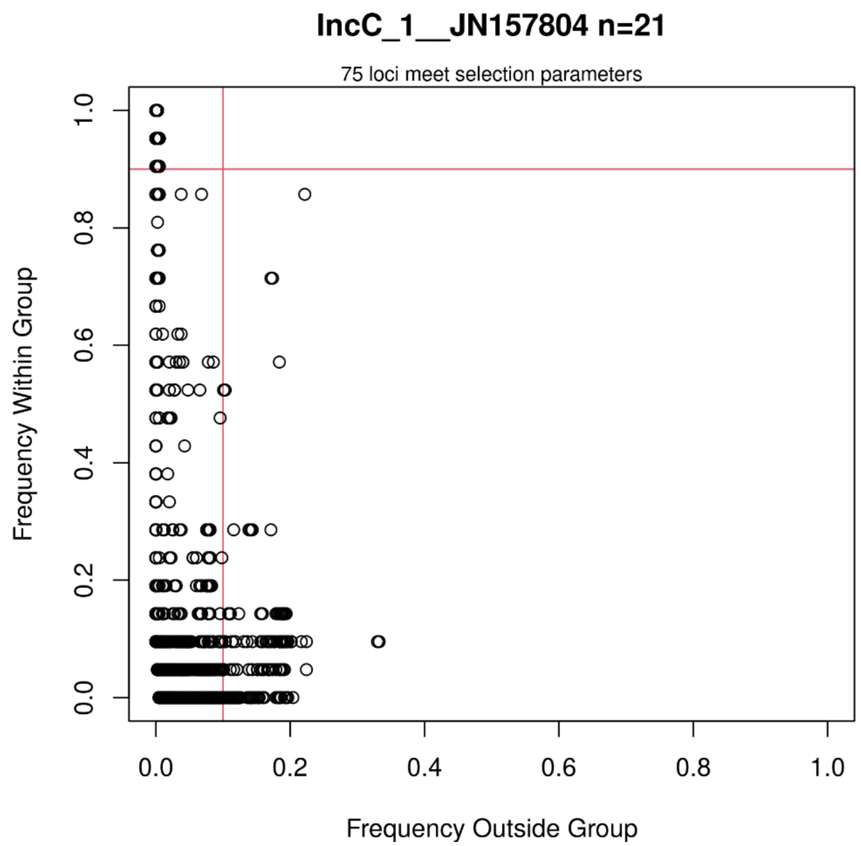


**Supplementary Figure 1**. A scatterplot comparing the prevalence of loci among 21 IncC plasmids (y-axis) to the prevalence among the 438 non-IncC plasmids in the dataset (x-axis). Red lines represent the user-defined prevalence cutoff values and the 75 loci in the upper-left quadrant meet or exceed the user-defined stringency criteria.

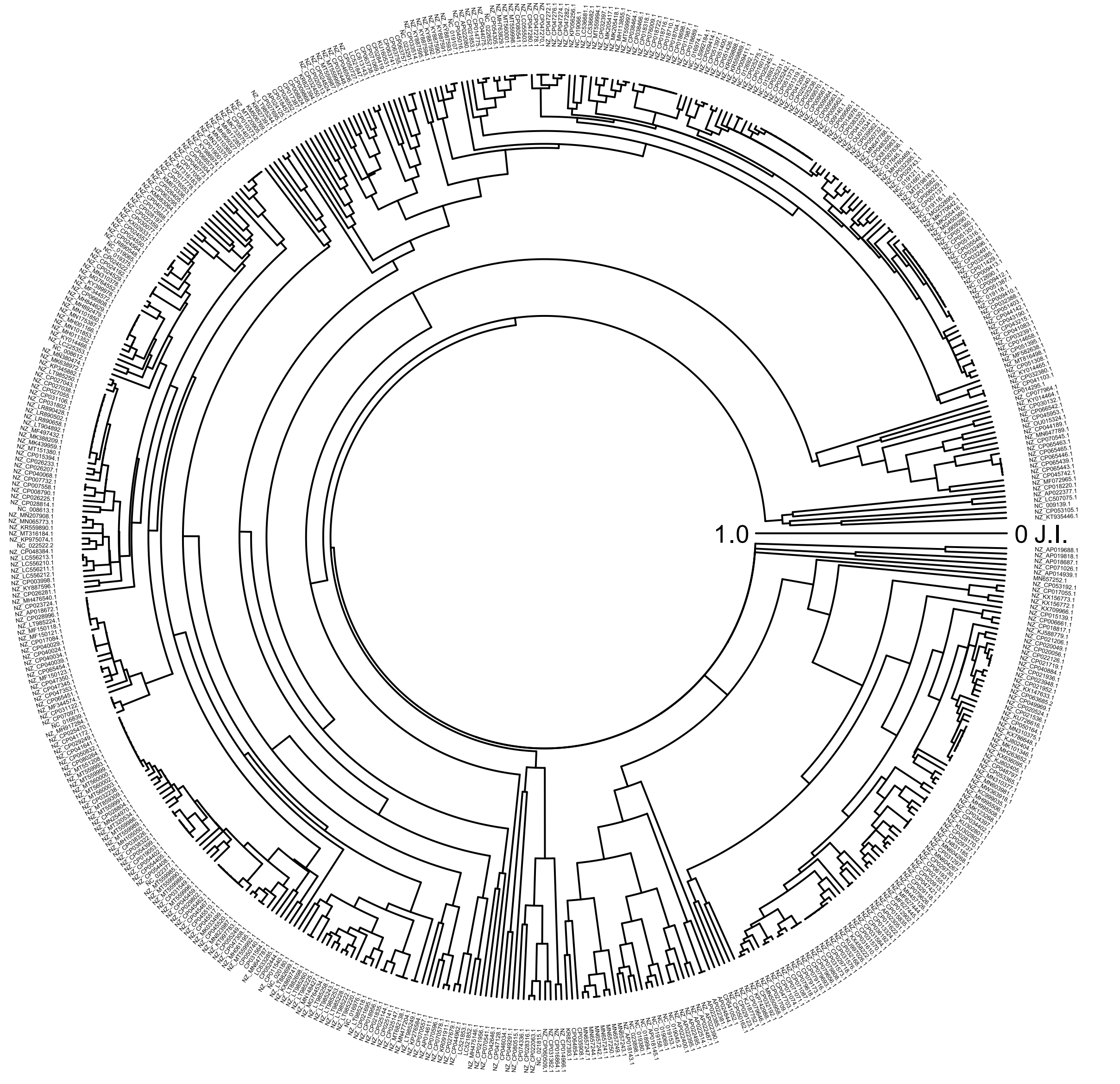


**Supplementary Figure 2.** Dendrogram of the 534 IncC plasmids from the PLSDB database constructed using the loci-allele patterns defined by the Lociq program. To account for difference in sequence length, duplicated and missing sequences, distances were calculated using the allele profile identifiers in stead of nucleotide sequence. Branch height corresponds to the Jaccard Index (J.I.) value.

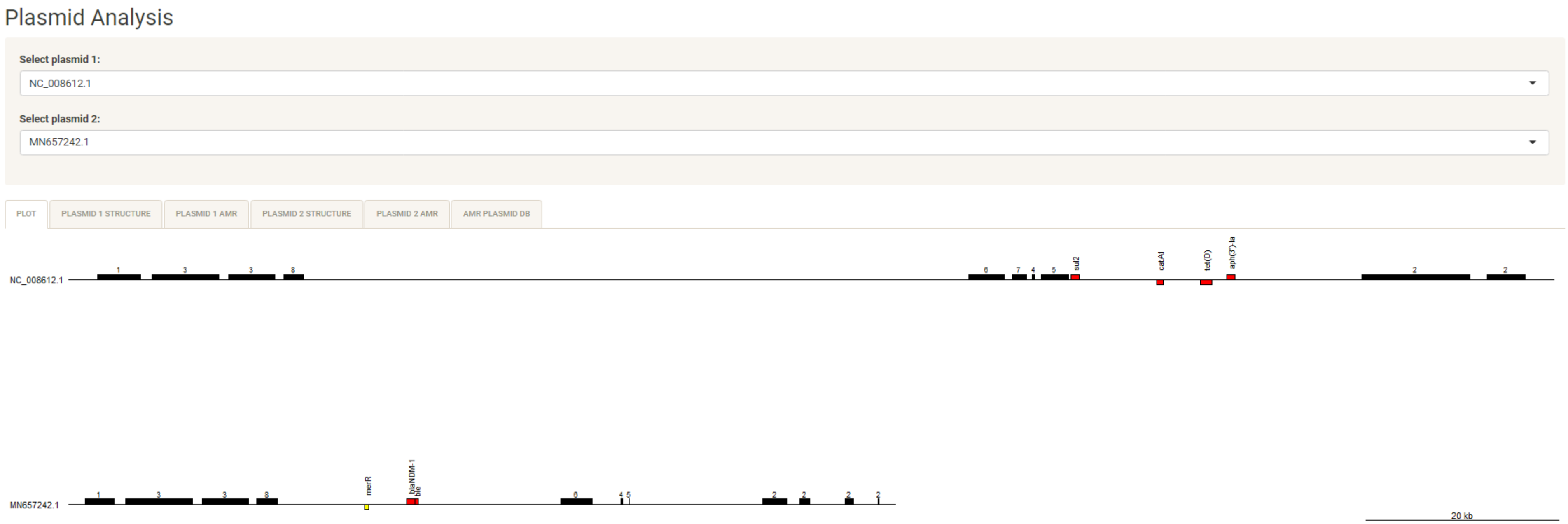


**Supplementary Figure 3**. Screenshot of the Lociq R-shiny application showing the graphical representations of the plasmids. Conserved IncC plasmid fragments are represented by numbered black bars, AMR genes by red bars and stress tolerance genes by yellow bars. User-selected plasmid pairs from the two drop-down menus are dynamically drawn to scale by the application and the screen contains multiple tabs for tabular descriptions of plasmid structural components, plasmid AMR genes and AMR composition of the full plasmid dataset.

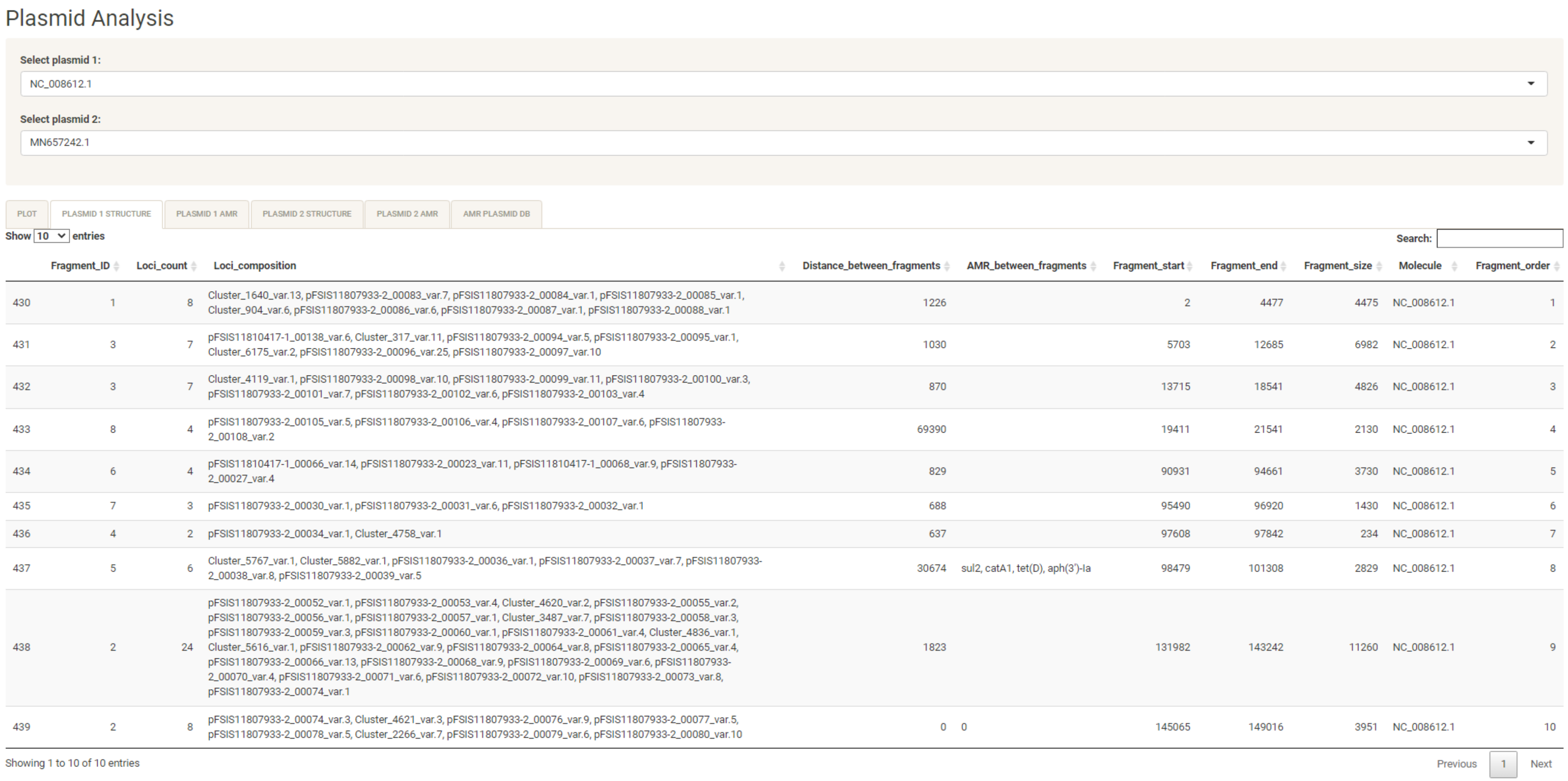

Plasmid Analysis

Select plasmid 1: NC_008612.1

Select plasmid 2: MN657242.1

PLOT | PLASMID 1 STRUCTURE | PLASMID 1 AMR | PLASMID 2 STRUCTURE | PLASMID 2 AMR | AMR PLASMID DB

Show 10 entries — Search:

| | Fragment_ID | Loci_count | Loci_composition | Distance_between_fragments | AMR_between_fragments | Fragment_start | Fragment_end | Fragment_size | Molecule | Fragment_order |
|---|---|---|---|---|---|---|---|---|---|---|
| 430 | 1 | 8 | Cluster_1640_var.13, pFSIS11807933-2_00083_var.7, pFSIS11807933-2_00084_var.1, pFSIS11807933-2_00085_var.1, Cluster_904_var.6, pFSIS11807933-2_00086_var.6, pFSIS11807933-2_00087_var.1, pFSIS11807933-2_00088_var.1 | 1226 | | 2 | 4477 | 4475 | NC_008612.1 | 1 |
| 431 | 3 | 7 | pFSIS11810417-1_00138_var.6, Cluster_317_var.11, pFSIS11807933-2_00094_var.5, pFSIS11807933-2_00095_var.1, Cluster_6175_var.2, pFSIS11807933-2_00096_var.25, pFSIS11807933-2_00097_var.10 | 1030 | | 5703 | 12685 | 6982 | NC_008612.1 | 2 |
| 432 | 3 | 7 | Cluster_4119_var.1, pFSIS11807933-2_00098_var.10, pFSIS11807933-2_00099_var.11, pFSIS11807933-2_00100_var.3, pFSIS11807933-2_00101_var.7, pFSIS11807933-2_00102_var.6, pFSIS11807933-2_00103_var.4 | 870 | | 13715 | 18541 | 4826 | NC_008612.1 | 3 |
| 433 | 8 | 4 | pFSIS11807933-2_00105_var.5, pFSIS11807933-2_00106_var.4, pFSIS11807933-2_00107_var.6, pFSIS11807933-2_00108_var.2 | 69390 | | 19411 | 21541 | 2130 | NC_008612.1 | 4 |
| 434 | 6 | 4 | pFSIS11810417-1_00066_var.14, pFSIS11807933-2_00023_var.11, pFSIS11810417-1_00068_var.9, pFSIS11807933-2_00027_var.4 | 829 | | 90931 | 94661 | 3730 | NC_008612.1 | 5 |
| 435 | 7 | 3 | pFSIS11807933-2_00030_var.1, pFSIS11807933-2_00031_var.6, pFSIS11807933-2_00032_var.1 | 688 | | 95490 | 96920 | 1430 | NC_008612.1 | 6 |
| 436 | 4 | 2 | pFSIS11807933-2_00034_var.1, Cluster_4758_var.1 | 637 | | 97608 | 97842 | 234 | NC_008612.1 | 7 |
| 437 | 5 | 6 | Cluster_5767_var.1, Cluster_5882_var.1, pFSIS11807933-2_00036_var.1, pFSIS11807933-2_00037_var.7, pFSIS11807933-2_00038_var.8, pFSIS11807933-2_00039_var.5 | 30674 | sul2, catA1, tet(D), aph(3')-Ia | 98479 | 101308 | 2829 | NC_008612.1 | 8 |
| 438 | 2 | 24 | pFSIS11807933-2_00052_var.1, pFSIS11807933-2_00053_var.4, Cluster_4620_var.2, pFSIS11807933-2_00055_var.2, pFSIS11807933-2_00056_var.1, pFSIS11807933-2_00057_var.1, Cluster_3487_var.7, pFSIS11807933-2_00058_var.3, pFSIS11807933-2_00059_var.3, pFSIS11807933-2_00060_var.1, pFSIS11807933-2_00061_var.4, Cluster_4836_var.1, Cluster_5616_var.1, pFSIS11807933-2_00062_var.9, pFSIS11807933-2_00064_var.8, pFSIS11807933-2_00065_var.4, pFSIS11807933-2_00066_var.13, pFSIS11807933-2_00068_var.9, pFSIS11807933-2_00069_var.6, pFSIS11807933-2_00070_var.4, pFSIS11807933-2_00071_var.6, pFSIS11807933-2_00072_var.10, pFSIS11807933-2_00073_var.8, pFSIS11807933-2_00074_var.1 | 1823 | | 131982 | 143242 | 11260 | NC_008612.1 | 9 |
| 439 | 2 | 8 | pFSIS11807933-2_00074_var.3, Cluster_4621_var.3, pFSIS11807933-2_00076_var.9, pFSIS11807933-2_00077_var.5, pFSIS11807933-2_00078_var.5, Cluster_2266_var.7, pFSIS11807933-2_00079_var.6, pFSIS11807933-2_00080_var.10 | 0 | 0 | 145065 | 149016 | 3951 | NC_008612.1 | 10 |

Showing 1 to 10 of 10 entries

Previous 1 Next

**Supplementary Figure 4**. Screenshot of the Lociq R-shiny application detailing the plasmid fragments from the first user-selected plasmid. From left to right, the columns display: master record row number, Fragment ID, number of loci on the fragment, the loci and allelic variants present on the fragment, distance between neighboring fragments, AMR gene content in the interfragment space, start position of the fragment, end position of the fragment, fragment size, current plasmid ID and the order that the fragment appears on the plasmid. Note that fragments may be divided into multiple parts if the plasmid typing loci are separated by a distance greater than the user-defined threshold.

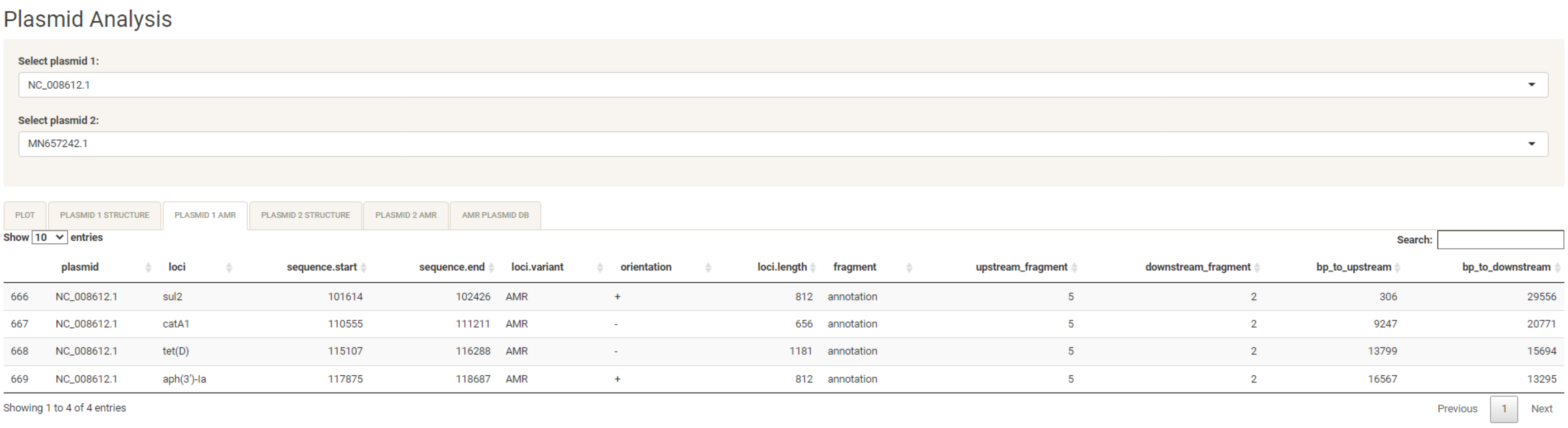

Plasmid Analysis

Select plasmid 1: NC_008612.1

Select plasmid 2: MN657242.1

PLOT | PLASMID 1 STRUCTURE | PLASMID 1 AMR | PLASMID 2 STRUCTURE | PLASMID 2 AMR | AMR PLASMID DB

Show 10 entries

Search:

| | plasmid | loci | sequence.start | sequence.end | loci.variant | orientation | loci.length | fragment | upstream_fragment | downstream_fragment | bp_to_upstream | bp_to_downstream |
|---|---|---|---|---|---|---|---|---|---|---|---|---|
| 666 | NC_008612.1 | sul2 | 101614 | 102426 | AMR | + | 812 | annotation | 5 | 2 | 306 | 29556 |
| 667 | NC_008612.1 | catA1 | 110555 | 111211 | AMR | - | 656 | annotation | 5 | 2 | 9247 | 20771 |
| 668 | NC_008612.1 | tet(D) | 115107 | 116288 | AMR | - | 1181 | annotation | 5 | 2 | 13799 | 15694 |
| 669 | NC_008612.1 | aph(3')-Ia | 117875 | 118687 | AMR | + | 812 | annotation | 5 | 2 | 16567 | 13295 |

Showing 1 to 4 of 4 entries

Previous 1 Next

**Supplementary Figure 5**. Screenshot of the Lociq R-shiny application detailing the position and neighboring fragment information for the AMR genes located on the plasmid.

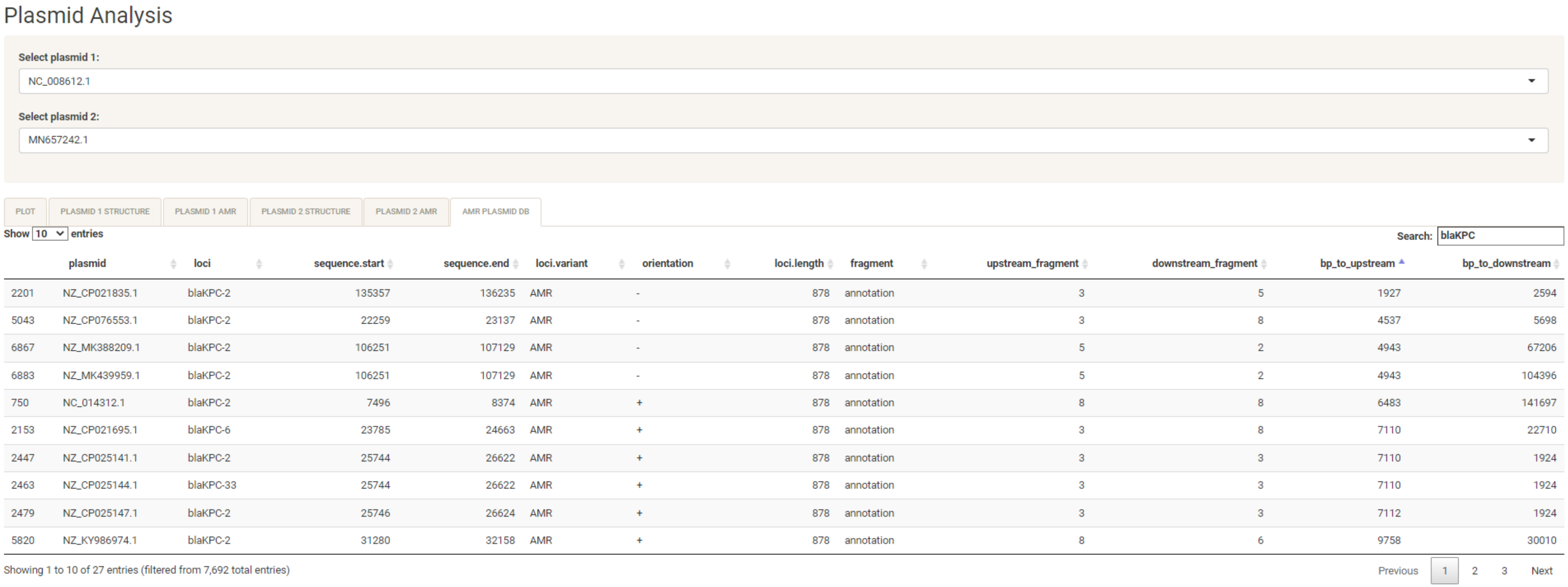


**Supplementary Figure 6**. Screenshot of the Lociq R-shiny application displaying a searchable datasheet for the AMR genes present in the plasmid dataset. This tab allows the user to filter the datasheet for plasmids that contain an AMR gene of interest (e.g. *bla*$_{\mathrm{KPC}}$ alleles) and evaluate differences in the position and neighboring fragments of the AMR gene between plasmids.

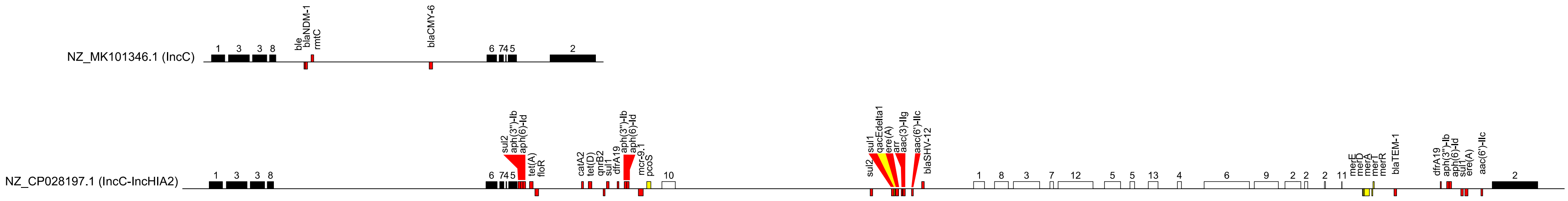


**Supplementary Figure 7**. Comparison of an IncC plasmid to an IncC-IncHIA2 hybrid plasmid. IncC fragments are represented by black bars and IncHIA2 fragments are represented by white bars. This diagram shows that the 5’ region of NZ_CP028197.1 is similar to the IncC plasmid NZ_MK101346.1. However, NZ_ CP028197.1 has a large region between IncC fragments 2 and 5 that contains plasmid fragments of the IncHIA2 plasmid type. The numbered black bars represent plasmid fragments, red bars represent AMR genes and yellow bars represent stress-tolerance genes. Strand orientation is in relation to the plasmid indexing locus and forward orientation is represented by gene presence above the sequence line.

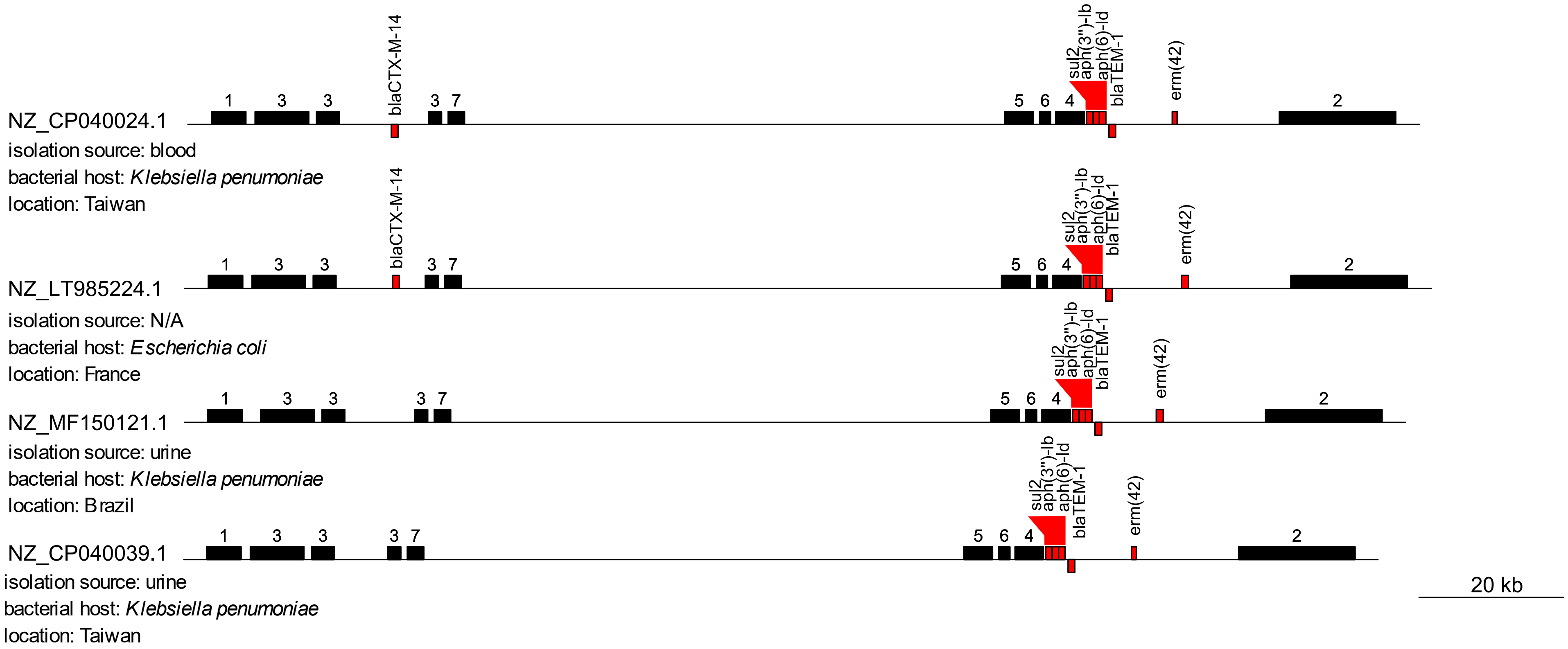


**Supplementary Figure 8**. A comparison of the IncC Lociq ST74 plasmids NZ_CP020024.1 & NZ_CP040039.1 to the IncC Lociq ST75 plasmids NZ_LT985224l1 & NZ_MF150121.1. The numbered black bars represent plasmid fragments and red bars represent AMR genes. Strand orientation is in relation to the plasmid indexing locus and forward orientation is represented by gene presence above the sequence line.

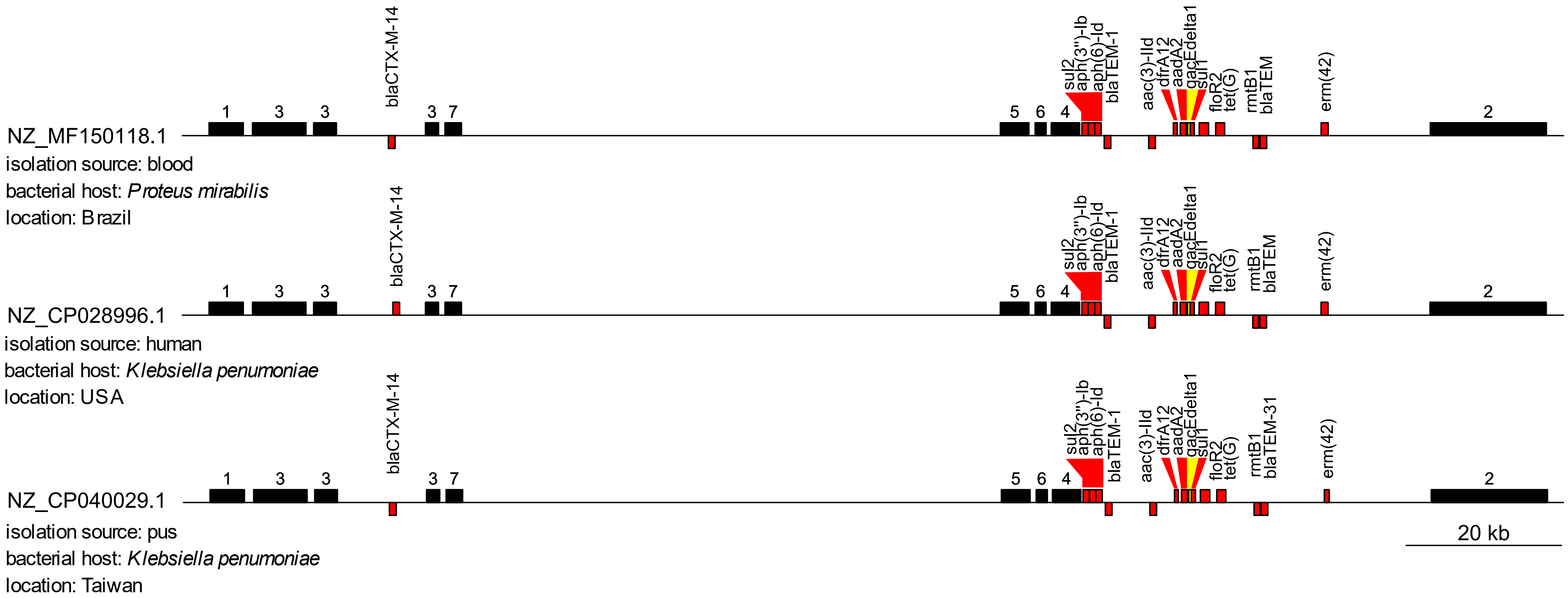


**Supplementary Figure 9**. A comparison of the IncC Lociq ST74 plasmid NZ_CP040029.1 to the IncC Lociq ST75 plasmids NZ_MF150118.1 & NZ_CP028996.1. The numbered black bars represent plasmid fragments, red bars represent AMR genes and yellow bars represent stress-tolerance genes. Strand orientation is in relation to the plasmid indexing locus and forward orientation is represented by gene presence above the sequence line.

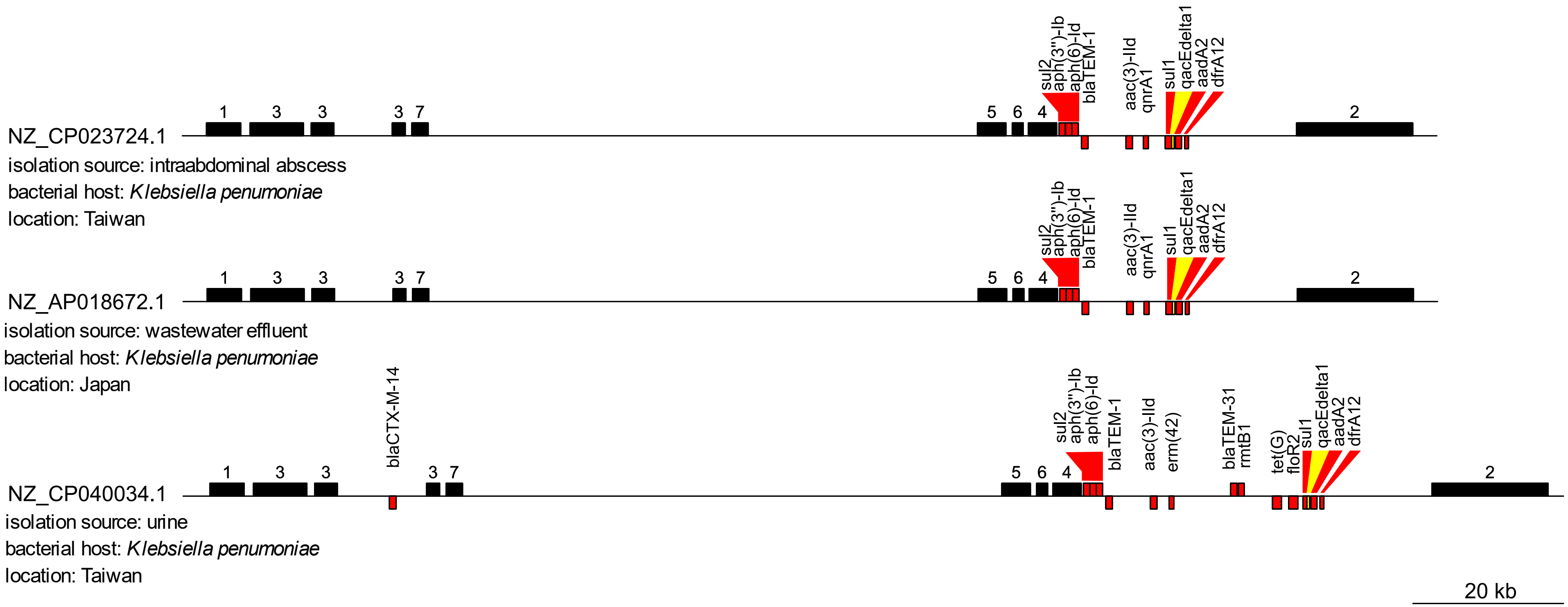


**Supplementary Figure 10**. A comparison of the IncC Lociq ST74 plasmid NZ_CP040034.1 to the IncC Lociq ST75 plasmids NZ_AP018672.1 & NZ_CP023724.1. The numbered black bars represent plasmid fragments, red bars represent AMR genes and yellow bars represent stress-tolerance genes. Strand orientation is in relation to the plasmid indexing locus and forward orientation is represented by gene presence above the sequence line.
