# Supplementary material for: Lociq provides a loci-seeking approach for enhanced plasmid subtyping and structural characterization
Source: Commun Biol. 2023 Jun 2;6:595. doi: 10.1038/s42003-023-04981-1 (PMC10238380; doi:10.1038/s42003-023-04981-1)
Supplement: Supplementary file 3 — Description of Additional Supplementary Files [file 42003_2023_4981_MOESM3_ESM.pdf]

Description of Additional Supplementary Files

Supplementary Data 1 - Plasmid Inc-type designations for the in-house plasmid dataset.

Supplementary Data 2 - Plasmid typing loci that were identified in the demonstration IncC dataset.

Supplementary Data 3 - A comparison of fragment sizes and the strength of correlation among loci to their cognate fragment with source data

Supplementary Data 4 - A Summary report of IncC plasmids from the PLSDB database using the IncC demonstration typing metrics

Supplementary Data 5 - Sequence definitions of plasmid alleles identified in the IncC demonstration dataset

Supplementary Data 6 - Sequence type definitions of the IncC plasmid fragments identified in the demonstration dataset

Supplementary Data 7 - Subgroups designations for plasmid typing method comparison

Supplementary Data 8 - Coordinate data for blaCMY-2 genes located between IncC plasmid fragments 6 & 8

Supplementary Data 9 - Partial loci that mapped to the end of a contig from the IncC demonstration set that were identified in the draft assembly JAANYS010000000

Supplementary Data 10 - Typing loci from the IncC demonstration set that may span contigs in the draft assembly of JAANY010000000

Supplementary Data 11 - Full length loci from the IncC demonstration set that were identified in the draft assembly JAANYS010000000
